# Practical training using immersive roleplay and an intensive course on clinical ethics consultation in Japan

**DOI:** 10.1186/s12910-022-00861-1

**Published:** 2022-11-24

**Authors:** Makoto Udagawa, Yoshiyuki Takimoto

**Affiliations:** Department of Biomedical Ethics, Faculty of Medicine, The University of Tokyo, Tokyo, Japan

**Keywords:** Clinical Ethics, Clinical Ethics Consultation, Role-play, Immersive role-play, Medical education

## Abstract

**Background:**

Clinical ethics consultation (CEC) is not sufficiently widespread in Japan. A possible reason is that a practical training system for CEC has not been established. Hence, we have developed “immersive role-play (IR)” as a practical training program that applies a new theatrical technique, immersive theater, to role-play learning. Its characteristics include high fidelity in the use of a studio and actors and dynamic realism in the progression of the scenario to immerse learners in role-play learning.

**Methods:**

We offered an intensive course on CEC for healthcare professionals from 2016 to 2019, with IR as part of the course curriculum. A questionnaire survey was administered to the participants regarding the educational effectiveness of IR. The questionnaire was anonymous, and participants responded on a 4-point Likert scale regarding their satisfaction with IR and their perception of its learning effects. An open-ended section at the end of the questionnaire asked about the effectiveness and areas for improvement of IR.

**Results:**

The questionnaire survey showed good results in all categories: usefulness, satisfaction, understanding, and interest. In the questions that compared the learning to traditional role-play learning, the participants gave significantly high ratings, specifically for “realism,“ “seriousness,“ “understanding the importance of communication skills,“ and “understanding the diversity of the narratives.“ In the free-text responses, the most frequent response was that they learned a lot about the practical procedures for participation.

**Conclusion:**

IR is sufficiently effective as a practical educational program, but not for everyone. It is suitable for those who are or will soon work as consultants. Conversely, beginners and intermediates who have not fully mastered the CEC theory and skills will need a stage-specific educational program separate from the IR.

## Background

The need for clinical ethics consultation (CEC) is widely recognized in increasingly complex medical settings. In Japan, the number of facilities providing CEC has been steadily increasing since the 2000s due to the influence of medical function evaluation and the strict guidelines set by the Ministry of Health, Labour and Welfare [1]. However, the number of CEC cases per year is only a few, thus making it difficult to conclusively determine whether effective CEC is still functioning in Japan [2]. The main reasons for this are the lack of a practical training system for CEC and the lack of a concrete CEC methodology [2].

Seminars on clinical ethics conducted in Japan are mostly short, with the main content being limited to desk-based lectures that (A) provide knowledge necessary for consultation through lectures and (B) develop advice and response skills for specific consultation cases through group discussions [3]. However, CEC is a practical activity that focuses on communication and requires the ability to respond flexibly in the clinical context. It is thus essential to learn specialized knowledge in classroom lectures, and cultivate skills to apply them and respond to various situations through practical training. Therefore, programs that focus on desk-based learning as described above are not sufficient; practical implementation is also required [4].

Of course, even though training in practice is essential, we cannot entrust inexperienced candidates with actual CEC practice as on-the-job training as CEC affects the patient’s treatment plan, and there is a risk of negative impact on the patient and medical staff in case there is any error in implementation. Therefore, as with clinical medicine, it is advisable to shadow learn CEC provided by experienced clinical ethics consultants on site. Nevertheless, this is where the difficulty lies in training CEC specialists in Japan. Considering that even large hospitals receive only a few requests for CEC each year, even if one were to go to a CEC facility for a month for training, the chances of actually witnessing CEC in action are infinitesimal, and even if one were to witness sparse cases, it would be an inadequate training opportunity [2]. Under these difficult circumstances, “how to secure opportunities for practical training with feedback and establish an effective training system” [5] has become a major issue for the training of CEC specialists in Japan. Simulation education, especially role-play training, is thought to be effective in ensuring practical training opportunities in CEC, where opportunities for clinical practice are scarce. The advantages of role-play learning include the ability to learn from mistakes without harming patients, the acquisition of skills, such as communication and situational awareness, the development of a proactive attitude and stance, and experiential learning, for situations that are difficult to practice in on-site training [6–10]. In contrast, problems with existing role-play learning have also been noted in prior studies. It is difficult to play the role in role-plays, mainly due to the lack of realism, and the role-play may become a game between novices. When a medical professional plays the role of a patient, he or she may not be able to fully assume the role of the patient, resulting in a lack of realism, thus hindering the scope of learning [11, 12].

Thus, in this study, we developed a new method, “immersive role-play (IR),” to overcome the above shortcomings of role-play in the clinical scenario. IR is a new theatrical technique known as immersive theater, applied to roleplay learning. Immersive theater is a theatrical technique in which “the audience walks of their own volition and participates in the work as part of the story, living together in the same space as the performers,” instead of the traditional “audience sits in the seats and watches the performers on stage” [13]. If we replace the audience with learners and the story with a clinical situation, the immersive theater becomes IR. The major difference between IR and previous role-play is a realistic clinical setting created by professional performers; this device immerses learners in the role of a clinical ethics consultant as part of a story that includes ethical dilemmas that can occur in the clinical setting.

In this study, we review the seminar in which we conducted IR, and describe the design principles, characteristics, implementation procedures, and scenarios of IR. Thereafter, we discuss its significance, considering the results of a questionnaire survey on IR.

## Details of the seminar

### Center for bioethics and law seminar

The Center for Bioethics and Law (CBEL) is a research and education center for bioethics and medical ethics in the Department of Biomedical Ethics, Graduate School of Medicine, The University of Tokyo. CBEL has been conducting seminars for medical professionals and medical students as outreach activities. This was Japan’s first attempt at an intensive seminar modeled after the intensive courses in biomedical ethics that are often offered in the U.S. The seminar was designed to provide students with a basic understanding of bioethics in a short period of time [14].

The CBEL Seminar began in 2004 and was suspended after 2019. The seminar consisted of a basic course (2 days) and four advanced courses: research ethics (1 day), clinical ethics (3 days), risk management (1 day), and public health ethics (2 days). The basic course and each of the four advanced courses were offered once per year, at different times. Only those who had taken the basic course could also take the advanced course. The inclusion of the CEC course is relatively recent. Pilot versions were conducted in 2009 and 2010, but due to staff shortages and other reasons, subsequent implementation was postponed. However, as the need for CEC began to be recognized in Japan, the CEC course was officially launched in 2016 with the full support of the Center for Patient Consultation and Clinical Ethics of The University of Tokyo Hospital, with a significant modification and reorganization of the pilot version of the curriculum with a focus on IR [14].

The following is an overview of the CBEL Seminar CEC course, which started in 2016 and ran for four years until 2019. The course aimed to provide primarily medical professionals with the specific methodology, knowledge, and skills needed to practice CEC, along with a theoretical background. The CEC course was largely divided into a theory section and practical section. The theory section addressed the knowledge and theory essential for CEC, assuming that the basic knowledge and skills (such as understanding “the four principles of biomedical ethics” and “the four quadrants approach”) had already been acquired in the basic course (refer to the next section for the contents of the theory section). The theory section did not resemble a complete classroom lecture, but incorporated group discussions and other work in almost all topics. Based on the theoretical part, IR was conducted in the practical part. The CBEL Seminar CEC course was a short intensive course lasting three consecutive days, with the first two days dedicated to theory, and the last day to practice—in 2016–2018, the program was held on three consecutive days, but in 2019, it was stretched to four days: two consecutive days for the theoretical part and two for the practical part. This was because each part of the program was held on a weekend to increase participation. The seminar ran from 9:00 AM to 6:00 PM on the day of implementation.

### Core competencies and curriculum

In designing a practical CEC course for the CBEL seminar, we began by identifying the core competencies of CEC in the Japanese context. The CEC core competencies themselves have been systematically presented by the American Society for Bioethics and Humanities (ASBH; 2011); thus, we used this as a basis for exploring competencies that fit the Japanese context. In the ASBH (2011), core competencies were broadly divided into (1) skills, (2) knowledge, and (3) attributes, attitudes, and behaviors [15]. Based on this, the CBEL Seminar CEC course classified core competencies into four categories: abilities, qualities, skills, and knowledge, as shown in Table 1, in accordance with the actual situation in Japan. When adapting the competencies to the Japanese context, we considered the fact that CEC was not widely used and understood in Japan, the strong authority of the attending physician in the Japanese medical field, the family-centered approach to decision making, and Japan’s unique view of life and death. Once the competencies were identified, we organized a curriculum for the development of those core competencies. We organized the program as shown in Table 2. Next, we briefly present how we view CEC in relation to the program.

**Table 1:** Core competencies of the CEC defined at the CBEL Seminar

Qualities	Sense of responsibility
	Courage
Ability	Insight
	Practical wisdom
	Sense of balance
Skills	Information-gathering skills
	Evaluation skills
	Analytical skills
	Solution-oriented skills
	Facilitation skills
	Communication skills
Knowledge	Knowledge of medicine
	Knowledge of ethics
	Basic knowledge of law
	Knowledge of psychology

(Qualities) In ASBH (2011), attributes, attitudes, and behaviors include patience, compassion, integrity, courage, and humility. We took “qualities” to broadly classify the aforementioned terms, and categorized them as shown in the table. Our addition of “qualities” was based on the situation in Japan at the time. That is, in 2016, when this course was conducted in Japan, clinical ethics consultation was expanding, but the existence of the clinical ethics consultant as a professional designation had not yet taken root, and professionalism (or professional virtue) needed to be taught.

(Ability) This item is not in the ASBH (2011). This is roughly equivalent to “Phronesis.”

(Skills) In ASBH (2011), core skills are divided into three major categories: skills to evaluate and analyze ethical issues, process skills, and communication skills. These include facilitation skills at conferences, skills for improving the quality of consultations, and for managing consultations. We have reorganized them into six categories as shown in the table, omitting those related to departmental administration and management, in light of the actual situation in Japan.

(Knowledge) In ASBH (2011), core knowledge was divided into nine categories, including moral reasoning and ethical theory, general bioethics issues and concepts, and healthcare systems. We categorized them into four major categories from a more practical perspective.

ASBH: American Society for Bioethics and Humanities, CBEL: Center for Bioethics and Law, CEC: clinical ethics consultation

**Table 2:** CBEL Seminar CEC course learning content, methods, and hours

Learning Content	Methods and hours
Knowledge CEC general theory (theory and methods) Theoretical background (procedural justice, autonomy, two-tier theory, virtue ethics, ethics of care, narratives, and decision-making) Psychological knowledge for communication Knowledge of the law Frequently consulted ethical issues Organization theory	Lecture, practice, and discussion (approx. 5 h) Conventional role play (approx. 4 h) IR (approx. 6 h)
Skills Information-gathering, information sorting, evaluation of information, analysis of ethical dilemmas, solving ethical dilemmas, determination of recommendations, tips to improve the effectiveness of CEC, communication skills, facilitation skills, reflection	Lecture, practice, and discussion (approx. 10 h) Conventional role play (approx. 4 h) IR (approx. 6 h)
Ability Sense of responsibility, courage	Conventional role play (approx. 4 h) IR (approx. 6 h)
Qualities Insight, practical wisdom, sense of balance	Conventional role play (approx. 4 h) IR (approx. 6 h)

CBEL: Center for Bioethics and Law, CEC: clinical ethics consultation

*“Conventional role-plays” are role-plays in which the participants play not only the role of the consultant, but also that of the doctor or other relevant person. It is a simple role-play exercise which aims to provide the participants with experience of information gathering and to provide feedback to each other on how to gather information

We adopted the ethics facilitation approach proposed by ASBH as the most effective method. In this approach, the consultant’s role is to gather and organize information, analyze the issues, and then support reasonable and ethical decision-making while helping to clarify the values of each person involved. Therefore, the seminar focused on acquiring the skills and knowledge needed to gather and organize information, understanding the value of each stakeholder, and supporting decision-making. In ethical analysis, we adopted a case-based approach originated by Johnsen, based on casuistry; we primarily used the four principles of medical ethics to identify problems. However, as there are many cases in which problems cannot be addressed solely by “specification” and “balancing” the four principles, we adopted a narrative approach based on narrative theory. This is a methodology that [a] considers differences of opinion (conflicts of values) as differences in the narratives, [b] sets a goal that can be shared by each stakeholder, and [c] attempts to solve the problem by proposing a new, acceptable narrative to reach that goal. In the theory section prior to IR, the aim was to learn these methods through lectures and exercises.

### On immersive role-play (IR)

#### Design policy of IR

There are many items to consider when practicing CEC: which parts of CEC to simulate in a CEC role-play—i.e., what learners will experience and learn—will depend on the readiness of the learners and the goals set for the course [7, 16]. It will also depend on the philosophical position of the CEC, such as how it is positioned as an activity. We designed IR as follows.

- Target learners.

The IR is not designed for beginners, but as a practical and effective exercise for experienced professionals who may be in a clinical setting in the future.

- Matters to be experienced and learned.

We designed the IR role-play with the intention of having the participants experience and learn the following points among the many features and cautions of CEC.

- Importance of Narrative
  One of the most important aspects of CEC practice is to be aware of the narratives of each person involved, to understand the ethical dilemmas in a case as narrative conflicts, and to seek to resolve them [17].
- Fragmentation of information
  In reality, when we try to understand narratives, we do not have all the information available to us at the outset, which makes it impossible to have a bird’s eye view of each person’s situation. Thus, consultants are required to infer and grasp the overall picture from the fragmented information obtained [18].
- Information fluctuations
  The information accumulated to understand the narratives of the people involved will also vary depending on the method and timing of the intervention. The consultant is also a member of the stakeholders when they participate in the project.
- Importance of communication
  The CEC is an entirely communicative activity. There are a variety of items on which to focus in each of the following areas: communication with the client, communication with stakeholders, and communication within the consultant team [19].

The above points can only be meaningfully experienced in a realistic and immersive environment. Therefore, in this study, we also worked to ensure that fidelity was high in terms of physical and environmental fidelity, patient and stakeholder fidelity, and learner psychological fidelity.

#### Implementation of IR

We conducted the IRs using the following procedure. Participants were limited to approximately 15 people per training session, who had received prior training in the theory and skills of CEC. They were divided into three groups (four to five participants per group), according to their work experience, and were asked to participate in the IR as a team. Each group was provided with a different IR scenario, and the other groups who were not conducting a simulated consultation were allowed to observe as spectators.

The role-play scenario began with a consultant receiving a request, during which, the participants as consultants, met with relevant parties and gathered information. After the scenario was completed, it was reviewed, during which the participants organized and evaluated the information, analyzed the problem, and summarized the recommendations. After the review, the role-play ended with the recommendation being communicated to the client.

There was approximately 30 min of pre-briefing for the role-play, 60 min for the scenario progression, 60 for review, and 60 for observation of other groups. Finally, the overall debriefing took approximately 150 min, making the overall program about 6 h long.

#### Preparation, stage, and staff of IR

**Fig. 1:**
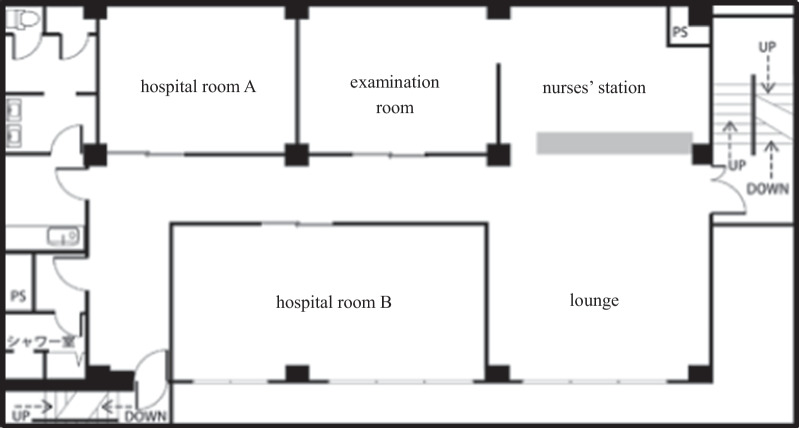
Stage of IR

The IR was held in a studio usually used for filming movies and TV dramas. The stage was composed of a nurses’ station, lounge, examination room, and hospital room, each including a simulated patient, doctor, nurse, and others (hereinafter referred to as “performers”). The stage was divided into sections, in which various events were performed simultaneously and in parallel (Fig. 1).

Each scenario involved eight performers, four of whom were professional actors. Among the performers, actors skilled in improvisation were assigned to important roles in the scenario, such as the client, the patient, and the doctor. The actors were asked to behave according to the scenario script at key points, but to be flexible in their communication with the learners playing the consultants, based on the characterization. Further explanation of what was required of the actors is provided below.

The IR was controlled by time, and events were supposed to occur to progress the scenario (story) at a defined time. We scripted a set of behavioral guidelines for these events and presented them to the actors. However, we asked that outside of the event, actors should respond to learners based on the characterization we had given the actors in advance. Although we prepared examples of standard responses, we instructed the learners to provide responses according to their attitudes. For example, if the scenario progression event was scripted as “the simulated patient will go to the doctor’s office at 2:00 p.m. for a meeting with the doctor,” then the actor needed to adhere to that event. However, the actor decided their attitude and mood when at the doctor’s office, and what kind of statements they would (or would not) make. The simulated patient, no matter how depressed or resentful they may have been due to the interaction with the learner, was expected to behave in a way appropriate to the situation to reach the doctor’s office at 2:00 p.m.

Table 3 summarizes the aforementioned “events that must occur” in chronological order and the “standard response” reference points. Depending on the role, for each of the performers, 80% of the hour-long role play was non-event time, or improvisation time that called for open performance. As mentioned above, IR has many demands on the actors, so rehearsals began approximately one month in advance.

**Table 3:** Actions of the main performers in Scenario 1 (excerpts from some of the characters)

			Patient		Patient’s partner		Attending physician	
Time		Major Events		Content/Scripted part		Content/Scripted part		Contents/Scripted part
8:40		Consulting request						
8:45		Move to the stage						
8:50	8:52	Declaration of refusal	Hospital Room B	Conversation with doctor Refusal of treatment			Hospital Room B	Doctor’s rounds Conversation with the patient
8:50	8:52					Come to the hospital and go to husband’s hospital room		
8:55	8:57	Patient’s roughness Wife’s exhaustion Nurses’ exhaustion	Hospital Room B	Refusing care from nurses Awkward silence	Hospital Room B	Harassing interactions between husband and nurse Awkward silence	Nr. St	Script [Ph Action 1].
9:00	9:02	Apology to medical staff Wife’s exhaustion	Hospital Room B	To his wife, “Leave me alone.”	Nr. St lounge	Leaves the HR and apologizes to the nurse at Nr. St. Moved to the lounge alone		
9:00	9:02	Head nurse and attending physician					Nr. St	Conversation with the head nurse
9:03	9:05	Differences in policies between doctors and nurses					Examination room	Conversation with the head nurse
9:05	9:10		Hospital Room B	Script [patient Action 1].	Lounge	Script [wife Action 1].	Examination room	Script [Ph Action 2].
9:15	9:17	Concerns about medical errors	Hospital Room B		Lounge	Conversation with the physician	Nr. St lounge	Found patient’s wife in the lounge, conversation
9:15	9:20	Patient’s true feelings, Nurse’s change of heart		Conversation with the head nurse				
9:18	9:22	Head nurse and patient’s partner	Hospital Room B	Script [patient Action 2].	Lounge	Conversation with the head nurse		Script [Ph Action 3].
9:25	9:30			Script [patient Action 3].		Script [wife Action 2].		
9:40	9:42	Physician’s hesitation	Hospital Room B	Script [patient Action 4].	Lounge	Script [wife Action 3].	Examination room	Conversation with the nurse
			Hospital Room B		Lounge	Script [wife Action 4].	Examination room	Script [Ph Action 4].
9:40	9:42	The dilemma of doctors and nurses	Hospital Room B	Script [patient Action ].	Lounge Hospital Room B	Script [wife Action 5] Return to HRB	Examination room	Conversation with the nurse
9:45	9:50	Briefing sessions, statement of refusal of treatment by the patient	HRB Examination room	Move to doctor’s office Briefing session begins	HRB Examination room	Move to doctor’s office Briefing session begins	Examination room	Briefing session begins
9:52	9:54	Head nurse and attending physician	Examination room	Return to Hospital Room B	Examination room	Return to Hospital Room B	Examination room	Conversation with the head nurse
10:00	10:02						Examination room	Call a consultant

There were 11 performers in Scenario 1, but the five people most involved with the CEC consultant were the patient, the patient’s partner, the attending physician, the nurse, and the head nurse (the consultant client). Detailed action charts were created for the afore-mentioned five persons, out of which three are described here. The performers were experienced actors and seminar staff (actual MDs and Ns) who had been training for several months

HRB: hospital room B, MD: medical doctor; N: nurse; Nr. St: nurse’s station, Ph: attending physician

#### Progression of IR

Learners in the consultant’s role first received a phone call from the performer playing the client role and were given an overview of the case. Then, working together in a group, they (1) gathered information by meeting the people involved in the five stages; (2) thereafter, they used this information as clues to understand the narratives of each person involved; (3) then, they had to identify the ethical dilemma in the given scenario; and (4) seek ways to resolve or eliminate it. (5) Finally, the process was completed by communicating the answers to the client.

All IR proceedings were controlled by time, and events were arranged to occur throughout the scenario. After the IR started with a phone call from the client, events defined in the scenario occurred somewhere on the stage according to time; and after a certain amount of time, the final process of the IR: the event of providing a response to the client occurred. The structure of the IR progression was such that the learner playing the role of consultant could not know about all the events that occurred in the scenario, nor could they fully grasp the changes in the feelings of the people involved. Three IR scenarios were prepared, all of which took approximately one hour to complete.

#### Features of IR

The researchers arranged for a studio, stage props, and actors to achieve high fidelity regarding the environment, the patients, and other people involved. This was intended to increase immersion and the psychological fidelity of the learner. However, IR encompasses much more than this. One of the crucial features of IR was that events occurred in each of the five sections that comprised the stage (examination room, nurses’ station, lounge, hospital rooms A and B) and proceeded simultaneously.

This had several advantages. First, in a real CEC scenario, it is impossible to obtain all the information at once, thus, making it necessary to understand the whole picture with limited information. The IR has difficulties like the inability (difficulty) to obtain information in parallel, and can be used to train inference, namely “to grasp the whole picture from fragments,” which is indispensable in real consultation scenarios. Furthermore, because IR involves simultaneous and parallel scenarios, the participants are required to work actively and efficiently together as a team. Additionally, because the situation changes with the learner’s intervention, the learner must become part of the story and carefully intervene with the people involved. Thus, IR participants are encouraged to participate more immersively in the role-play as “one of the people involved” because of this dynamic mechanism of scenario development.

#### Evaluation of IR

In the IR, learners playing the role of consultants were evaluated by scorers on how well they performed their tasks. Scorers were placed at various locations on the stage. Performers also participated in the evaluation as commentators.

The evaluation was divided into individual and group evaluation items. The individual evaluation items were divided into four items for communication skills and two for commitment. The group evaluation items included three items that questioned the level of achievement in understanding medical facts, understanding the narratives of the people involved, and the effectiveness of problem-solving, including an item that questioned about collaboration within the team. All 10 items were rated on a 3-point scale, after which an overall rating (3-point scale) was determined. Evaluation sheets and performer comments were collected for each scenario, and an evaluation meeting was held after the completion of all scenarios.

#### Scenarios of IR

The following three scenarios were created by us as scenarios of IR, which were based on the theme of refusal of treatment. There are two main reasons why we created three scenarios on the theme of refusal of treatment. The first is because treatment refusal is often seen in Japanese clinical practice (against the background of Japanese cultural circumstances that differ from Western self-determination centrism) [20, 21]. Second, closely related to the first, is to contribute to the learner’s future problem-solving methods in the casuistic approach. In casuistry, cases are accumulated, and for difficult cases, solutions are sought by comparison and analogy with other cases that have been resolved [22]. The accumulation of ethical considerations of similar cases of refusal of treatment will be a great asset for future learners.

Although all of the scenarios focused on the theme of refusal of treatment, the background and reasons for the refusal were very different, and in all scenarios, the learner was required to understand the narratives of the people involved and to resolve the conflicts (Table 4).

**Table 4:** Scenario outlines

Scenario 1: Refusal of treatment stemming from being mentally trapped
Time span: approximately 1 h Client: Head Nurse This case involved consultation on how to deal with a 35-year-old male patient on dialysis due to diabetic nephropathy. The patient was hospitalized for a shunt reconstruction, but he became depressed and mentally trapped by his situation and started refusing to receive catheter dialysis. Moreover, he was also causing problems by verbally abusing his wife and nurses. The assignment of the consultation was to determine whether it is ethical to recommend discharging the patient from the hospital if he refuses to receive shunt revision surgery.
Scenario 2: Refusal of treatment for religious reasons
Time span: approximately 1 h Client: Nurse in-charge This case was a consultation regarding the treatment of a 40-year-old female patient admitted to the cardiology department for pulmonary hypertension. The patient needed a percutaneous cardiopulmonary support system (PCPS), but she refused it for religious reasons. However, the attending physician did not care about the patient’s wishes and tried to obtain informed consent from the family to proceed with PCPS because the patient was also being treated for schizophrenia. The details of consultation were regarding what steps to take to be there for the patient.
Scenario 3: Refusal of standard treatment stemming from an attitude of avoiding self-determination
Time span: approximately 1 h Client: Attending physician (gastroesophageal surgery) This case was a consultation on how to deal with a 70-year-old female patient who was hospitalized for stomach cancer. The patient had started to avoid making decisions on her own after her husband’s suicide, and has refused the standard treatment proposed by her doctor for her stomach cancer, following the advice of her eldest son, who recommends non-standard treatment. The patient’s only stated wish was that she did not want to be transferred to a different hospital. The assignment of consultation was how to deal with the patient’s reluctance to be transferred to a different hospital.

## Methods

A questionnaire survey was conducted on the educational effectiveness of IR for participants in the CBEL Seminar CEC course, which was conducted over a four-year period from 2016 to 2019. The participants were exclusively healthcare professionals, totaling 75 participants over the four-year period (no duplicates). The questionnaire was administered after the entire CBEL Seminar CEC course was completed each year. The responses to the survey were recorded on a 4-point Likert scale. The question items addressed satisfaction with IR and perceived learning effectiveness; the responses were recorded anonymously. The answers were simply tabulated and analyzed using descriptive statistics. An open-ended response section was also included at the end of the questionnaire, asking about the effectiveness of IR and identifying the areas for improvement. The number of responses is shown in Table 5.

**Table 5:** Number of open-ended responses

2016	On effectiveness	16
	On improvements	7
2017	On effectiveness	14
	On improvements	10
2018	On effectiveness	15
	On improvements	9
2019	On effectiveness	17
	On improvements	7

After tabulating the data, the responses to the open-ended questions were coded for content analysis. One of the authors labeled the responses, the other reviewed them, and finally, the two authors discussed and decided on the codes.

## Results

A total of 75 participants attended the four IR sessions, 62 of whom had previous experience with role-play learning (Table 6). The total of “Strongly agree” and “Agree” responses to the question about “useful in practice” in the IR was 100%. The response was also extremely positive, with 99% satisfaction with the exercise, 100% for deeper understanding of CEC, and 100% for increased interest in the practice of CEC (Table 7).

**Table 6:** Experience of the participants in role-play learning

Occupations of participants (n = 75)		
Physician	37	49%
Nurse	36	48%
Other	2	3%
Experience in role play learning		
Yes	62	82.6%
	Physician	41.3%
	Nurse	41.3%
	Other	0%
No	13	17.3%
	Physician	8%
	Nurse	7%
	Other	3%

**Table 7:** Responses of the participants (n = 75)

Items	Strongly agree		Agree		Disagree		Strongly disagree	
I think IR would be useful in the practice of clinical ethics consultation	60	80%	15	20%	0	0%	0	0%
I was satisfied with IR	54	72%	20	27%	1	1%	0	0%
I have a deeper understanding of CEC through IR	58	77%	17	23%	0	0%	0	0%
I have increased interest in the practice of CEC through IR	57	26%	18	24%	0	0%	0	0%

The 62 participants who had experienced regular role-playing in the past were asked to compare IR with the role-playing they had experienced in the past (Table 8). Overall, IR received better responses than previous role-play learning, but the superiority of IR was particularly pronounced with regard to the following four areas: “realism,” “seriousness,” “understanding of the importance of communication skills,” and “understanding of the diversity of narratives.”

**Table 8:** Comparison of IR and previous role-plays (n = 62)

	Strongly agree		Agree		Disagree		Strongly disagree	
IR was more realistic	50	81%	11	18%	1	**2**%	0	0%
It was easy for me to play the role	34	55%	23	37%	5	8%	0	0%
I could seriously participate in IR	50	81%	10	16%	2	3%	0	0%
It was easy for me to actively participate	41	66%	15	24%	5	8%	1	2%
I was able to recognize the importance of communication skills	45	73%	14	23%	1	2%	1	2%
I was able to understand that there are various narratives in the clinical setting	48	77%	11	18%	3	5%	0	0%

Coding of the open-ended responses regarding effectiveness (62 responses) showed that the largest number of responses (32) fell into the “practical and procedural” category, followed by “realistic” (20), “information gathering” (16), “overview and reflection” (13), “virtues, motivation, and qualities” (10), “narrative” (10), and “teamwork” (8) (Table 9).

**Table 9:** Open-ended responses regarding effectiveness and improvements

Label	Number of labels	Examples
*Open-ended response regarding effectiveness*		
Practical and procedural	32	“I think this training has taught me procedures that I can use in my practice.” “I have just started consulting, but I feel like I can take the training wheels off my bike.”
Realistic	20	“It was very realistic and hands-on, so I felt it was easy to put into practice at the clinical site.” “It was very helpful because I did not have concrete experience of CEC.”
Information gathering	16	“It helped me to collect information in a civilized manner.” “I realized how difficult it is to gather information and to grasp the whole picture.”
Overview and reflection	13	“It was an opportunity for me to look at how others see me objectively. It is better to be an observer than to perform the role myself, so I can get a bird’s eye view.” “It was just like a real clinical practice, so I was able to learn about my own habits and how to behave. I learned a lot from watching other people’s movements in the audience.”
Virtues, motivation, and qualities	10	“I am sure I will gain some confidence through the experience.” “It inspired me to be brave and go into the clinical field!”
Narrative	10	“It was helpful to understand the various narratives that exist behind the scenes and how to conduct them in the future.” “I now understand the significance of confirming the situation and feelings from as many people as possible, as well as the limitations of doing so.”
Team-work	8	“It was meaningful in that it reaffirmed the importance of teamwork.” “As we had not been working as a team, we were able to realize the difficulties and advantages of working together as a team.”
*Open-ended responses regarding improvements*		
Need more time for IR	12	“Wish there was more time for consideration.” “Wish we could have taken a little more time.”
Need more information in advance	5	“I thought it would have been easier to immerse myself in the role play if the background of the ethics consultation team and the backgrounds of its constituent members had been predetermined.” “I do not have basic knowledge of the disease, so I need time to research it. I thought it would be better to have some information about the direction and content of the treatment in advance.”
Space and venue improvements	2	“Nurse’s station should be a little wider.” “Would have been nice to have a conference room. Hard to consider.”
Schedule	2	“I wish the CEC course implementation dates had been set earlier.”
Observer	2	“I felt there were a few too many observers.” “It would have been nice to have something to do while I was observing.”
Tools	1	“I wish my cell phone (PHS) could have been linked.”
Evaluation	1	“I wanted the staff to evaluate each action to see if it was good or not.”
Cost	1	

Regarding what they learned in the IR, many respondents gained insight into practical ways of how to proceed with CEC, how to collect information, and communicate with others (“I think I learned procedures that will be useful in practice” and “It was very helpful for me to collect information in the future”). However, many also mentioned the importance of working in teams (“I was able to understand specific points to keep in mind when working in teams”), the difficulty of grasping the whole picture from fragmented information (“I realized how difficult it is to collect information and grasp the whole picture”), and the importance of grasping narratives (“I could see that there are various narratives behind the scenes, and it was helpful for me to understand how to do things in the future.”). Many participants also stated that they were able to view CEC from a different perspective through IR, stating, “It gave me an opportunity to objectively reflect on how I behave and how I am viewed by others.”

The most common free response regarding improvements was “I would like more time for IR.” The next most common response was related, namely “I would have liked more advance information about the cases.” Other requests were for improvements in facilities and scheduling, but there were also requests for improvements in evaluation (“I wanted the staff to evaluate each action to see if it was good or not”).

## Discussion

The simulated ethics consultation exercise using IR received very good responses in all areas of “usefulness,” “satisfaction,” “understanding,” and “interest.” It also received adequate responses overall when compared to traditional role-play learning. In particular, “realism,” “seriousness,” “understanding of the importance of communication skills,” and “understanding of the diversity of narratives” received high evaluations. Furthermore, the open-ended responses revealed that many participants learned about practical procedures. These results may indicate that we were able to create a successful IR environment and that all of the four IR experience/learning objectives presented in Sect. 2.3—(a) importance of narrative, (b) fragmentation of information, (c) variability of information, and (d) importance of communication—were fully achieved.

### Design policy and educational effectiveness

The most common CEC exercise is the examination of a case on paper [23, 24]. In this exercise, from a pre-prepared case outline, the participants organized the situation and identified the ethical dilemma presented in the scenarios. The information necessary to understand each person’s narrative is already objectively presented all at once on paper. The advantage of this exercise is that it is very easy and can be performed anywhere, and it has some effectiveness as an exercise focused on the analysis and examination of a problem [25]. However, as all the information is presented at once, it does not allow for gradual training, whereby the whole picture has to be inferred from fragments of information. There are also some drawbacks, such as the lack of opportunities to communicate with the people involved in obtaining the information, and a lack of a sense of realism because the information is presented in objective descriptions [26].

In normal role-plays, increasing environmental and physical fidelity and patient fidelity can make learners aware of the importance of communicating with the people involved to obtain information and increase their awareness of being involved as a member or a stakeholder [6–9]. Furthermore, the shortcomings of paper exercises can be overcome to some extent with this normal role-play exercise. However, there are limitations in a classroom setting with strict time and human resource constraints.

The IR is an attempt to overcome such limitations. It takes environmental and physical fidelity to the extreme and runs scenarios simultaneously and in parallel, thus realizing information unavailability due to time and space limitations. This enables training in which “the entire picture is gradually inferred from the accumulation of fragmentary information.” Moreover, by increasing patient fidelity to the utmost limit, we encouraged the participants to be aware that they were intervening as a concerned party. Compared to paper exercises and traditional role-playing, IR enables practical training by increasing environmental and physical fidelity, as well as patient fidelity, thereby preventing the learner from having a bird’s eye view of everything.

Considering these characteristics, IR is not an exercise for beginners, but for those with experience who may be currently in clinical practice. The practical and effective educational effects of IR are well expressed in the results of the questionnaire. It could be argued that the IR, as originally designed, provided meaningful experience and confidence to those who had learned the basics of CEC but had no clinical experience or were inexperienced as consultants.

### Effectiveness of the audience role system

One of the unique features of IR is the “Audience” system, in which individuals can participate not only as consultants and conduct role-plays, but also as spectators if they are not the consultant (if another team is playing the consultant role). In IR, the learner in the audience role is treated as “non-existent” by the consultant role learners and performers, and are free to move about the stage, listen to other people’s conversations, and view medical records at the nurse’s station. In IR, the learner in the consultant role is expected to gather information and understand each person’s narrative by actively contacting the relevant parties on their own. Learners in the audience role can learn from a bird’s eye view of how to gather information and grasp narratives through observing learners in the consultant role from the privileged position of being treated as a “non-existent person.” Indeed, some of the open-ended responses included the following: “It is better to be an observer than a performer (learner) to get a bird’s eye view,” and “By playing the roles of exerciser and spectator, I could also see the big picture.”

Immersion and becoming part of the story alone diminishes its significance as a role-play and immerses the learner in the situation. As it is a role-play, we believe that it is also important to be an audience member who has a bird’s-eye view of the story’s progression. There are two main reasons for this—one is that it is only from the non-restrictive position of the audience that one can better understand the limitations of the consultant’s restricted position as one of the parties involved. Second, to be an audience is to engage in observational learning. As indicated in the open-ended responses, learners gained a variety of insights by observing other learners’ CEC as a member of the audience (“It was good to experience the difficulty of actual communication, but even better to observe other people communicating” and “I learned a lot from watching other people’s movements in the audience”) [27, 28].

### Cultivating qualities

In CEC, it is often difficult to find the absolute correct answer to a given scenario. It is impossible to gain a “God’s perspective” on the information and on how to analyze and solve problems. Even the participants are able to gather information, analyze the problem, and find a solution without fault, the result may nevertheless be negative. This differs from an exercise in which a model solution is provided in advance. In the actual CEC, difficulties have to be addressed in a limited amount of time, under immense pressure.

What is important in the actual CEC described above is to understand and admit that it is difficult to know which is the correct answer in a given scenario in a clinical setting, and to make the correct choice despite the difficulties. Professional virtues, such as integrity, courage, and a sense of responsibility (“qualities” in our classification of core competencies) are required to cope with such precarious situations in clinical practice. The opportunity to enhance one’s professional virtues (qualities) can also only be obtained in a clinical setting. However, if IR enhances the sense of immersion, deepens the awareness of being part of the story, and increases learner fidelity, it may be possible to experience the “consultant’s limitations” and enhance virtues (qualities) to some extent. Indeed, some of the open-ended responses included comments such as “I think it will inspire me to be courageous in the field,” and “I am humbled by the experience.”

### Limitations and improvements of IR

One of the advantages of the role-play technique is that it provides an opportunity to switch roles and take on a different position easily [29]. For example, for the physician, taking the patient’s place can be a very important opportunity to reflect on his or her own behavior as a physician; in IR, the simulation is designed to maximize the fidelity of the actor, including patient fidelity. Therefore, it is not possible to have the learner also play the patient, as is usually done in a role-play. Thus, one might say that IR lacks the major advantage of role-shifting. However, IR has an audience system, which compensates for “seeing from another perspective” to some extent, although not to the extent of role-shifting. In fact, one of the comments in the open-ended responses was “It gave me an opportunity to objectively reflect on how I behaved in order to see how others saw me. It is better to be an observer than a performer to get a bird’s eye view of the entire scenario.”

Moreover, IR is intended for those who may be immediately commissioned as consultants. Therefore, it is not suitable for beginners and intermediate students who have not yet fully learned the theory and skills of CEC. Simulation exercises (e.g., on-paper exercises and regular role-plays) are thus required for beginners and intermediate students according to the learning objectives of each level.

Furthermore, IR is characterized by long duration, which makes it difficult to ensure sufficient time for debriefing. Debriefing is a crucial step in role-playing [29, 30]. In our program, we set the debriefing time at 150 min, but for longer role-plays such as IR, it may be necessary to take more time to reflect on the debriefing, which raises the concern that sufficient feedback was probably not provided to each learner.

### Limitations of this study

In this study, the number of participants who experienced IR was small; thus, we were unable to obtain a sufficient number of surveys regarding its educational effects. Hence, the analysis was limited to descriptive statistics. In addition, due to the small number of subjects, it was not possible to ask about the job title, years of experience, and the size and type of medical institution where the participants worked, to eliminate the risk of being identified.

There are also limitations in terms of evaluation. One such limitation is that we were unable to measure the learning effects of IR based on objective indicators. In our IR, the final evaluation of an individual learner was determined by combining the evaluation of the individual learner (6 items) and the evaluation of the group to which the learner belonged (4 items), but we were unable to examine the effectiveness of IR extensively by comparing the individual learner’s evaluation and the group’s evaluation. This is an issue that should be addressed in future work.

Furthermore, it cannot be ruled out that the format of the event in 2018 was slightly different from other years, which may have affected the results of the survey. Moreover, it is unknown the extent of the behavioral change IR actually fosters in CECs and how long the effect lasts. In the future, we would like to secure a sufficient sample, measure the educational effects based on objective indicators, and investigate the behavioral changes before and after the IR experience to gain in-depth knowledge.

## Conclusion

In this study, we designed and implemented IR as a practical and effective CEC role-play. The IR maximized environmental and patient fidelity and enhanced the immersive experience of the participant by facilitating concurrent and parallel scenarios. The purpose of IR is to have learners experience and learn the following: (a) importance of narrative, (b) fragmentation of information, (c) information fluctuations, and (d) importance of communication.

For this study, the IR was conducted from 2016 to 2019 and participants were surveyed and found to have positive responses in all areas of usefulness, satisfaction, understanding, and interest. The overall response was also better than that of traditional role-play learning. In particular, the “realism,” “seriousness,” “understanding of the importance of communication skills,” and “understanding of the diversity of narratives” received high evaluations. Furthermore, the open-ended responses revealed that many learned about practical procedures, which might be beneficial to them in the future. The audience system was also shown to be effective. These results indicate that the experience and learning were realized as we intended, and that the IR functioned as a program with sufficient educational impact.

## Data Availability

The datasets generated and analyzed during the current study are not publicly available because consent for data sharing was not obtained from the participants, but are available from the corresponding author on reasonable request.

## Abbreviations

CEC: Clinical ethics consultation
IR: Immersive role-play
CBEL: Center for Bioethics and Law
ASBH: American Society for Bioethics and Humanities
